# Toxic epidermal necrosis associated with afatinib: A case report and literature review

**DOI:** 10.3389/fonc.2022.1010052

**Published:** 2023-01-09

**Authors:** Wei Yang, Yansong Lu, Ze Wu, Jun Niu

**Affiliations:** Department of Dermatology, General Hospital of Northern Theater Command, Shenyang, China

**Keywords:** afatinib, tyrosine kinase inhibitor, non-small cell lung cancer, cutaneous adverse drug reactions, toxic epidermal necrolysis

## Abstract

**Objective:**

To report a case of afatinib-induced toxic epidermal necrosis (TEN), in a patient with metastatic non-small cell lung cancer (NSCLC) and compare these findings with that of evaluate similarities and differences to other cases reported in the literature.

**Methods:**

With use of the algorithm of drug causality for epidermal necrolysis (ALDEN), the effects of afatinib were evaluated in a NSCLC patient who developed TEN. In addition, previous case reports on this topic were included to provide a review of patients’ clinical characteristics, treatment regimens and therapy outcomes in response to afatinib treatment.

**Results:**

In our case, toxic epidermal necrolysis was observed at five days after afatinib therapy, while other Stevens-Johnson syndrome/toxic epidermal necrolysis responses, as associated with afatinib, did not seem to be induced until a latency period of over thirty days post-afatinib. Treatment with corticosteroids resulted in significant improvements of these clinical symptoms, and eventually to a complete remission.

**Conclusion:**

Afatinib can result in grade four cutaneous adverse effects like SJS/TEN, with an uncertain latency period. The skin lesions which appear during this period of afatinib treatment should be closely monitored.

## Introduction

Stevens-Johnson syndrome/toxic epidermal necrolysis (SJS/TEN) is an infrequent, but severe adverse drug reaction. SJS/TEN can be considered as being on a continuum of disease spectrums with <10% of the body surface area affected in SJS, >30% in TEN, while 10-30% of the body surface area affected in the SJS-TEN overlap (1). The typical manifestation of these lesions, as detailed in the medical history and biopsy of the patient, are critical in confirming this diagnosis.

In patients with metastatic non-small cell lung cancer (NSCLC) one of the possible first-line treatment options is afatinib (2), which is a small molecular tyrosine kinase inhibitor that inhibits tyrosine kinase activity of the epidermal growth factor receptor (EGFR). However, afatinib can be associated with adverse skin-related reactions, including acne, xerosis, paronychia and purpuric rash (3). To our knowledge, only a few case reports of afatinib-induced SJS/TEN exist in the literature.

## Case presentation

**Figure 1:**
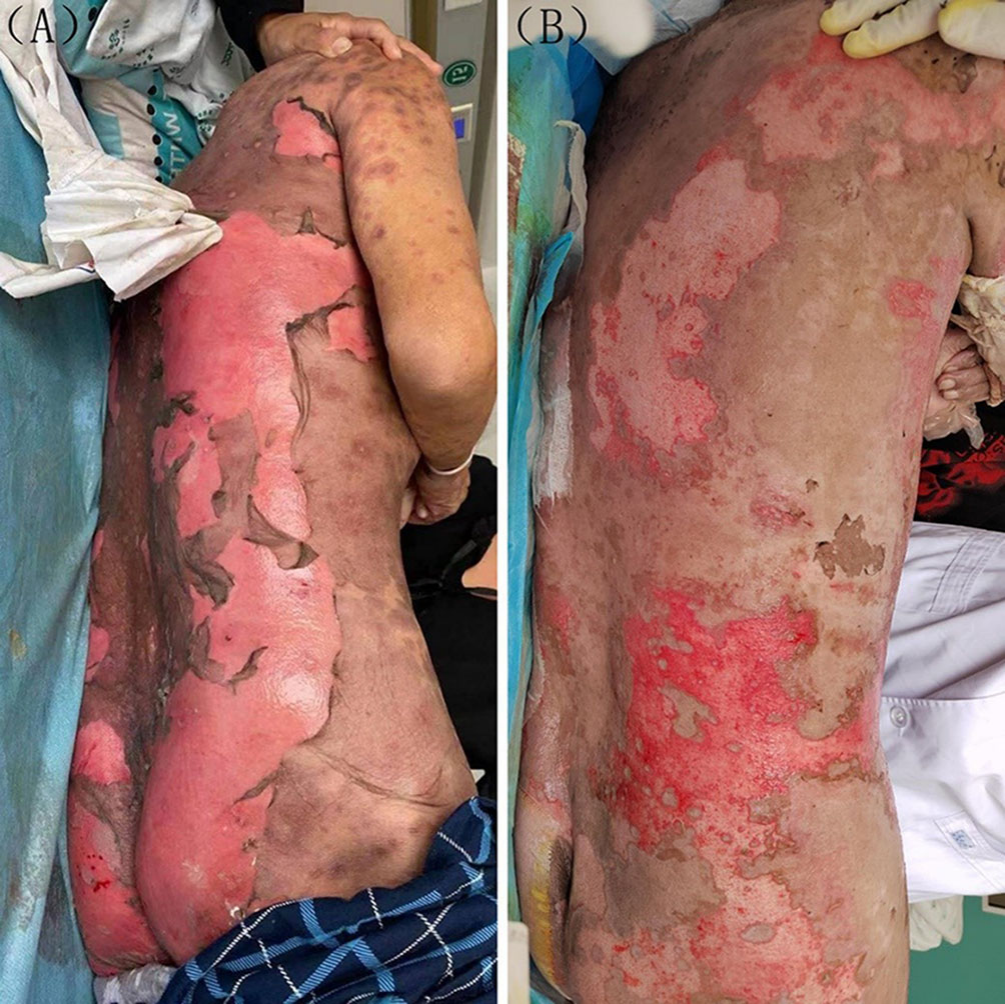
**(A)** Before treatment large sheets of necrolytic epidermis exfoliated are present on the back. **(B)** After one week of treatment, there was a partial improvement in the cutaneous lesions.

A 62-year-old Chinese male was diagnosed with right lung adenocarcinoma involving brain and bone metastases in August of 2021. Treatment consisted of a daily oral administration of 40 mg afatinib. Five days after receiving afatinib, erythema and blisters were observed on the trunk and limbs along with an increase in body temperature to 39.0°C. Ruptured bullae and skin exfoliation gradually appeared within the following two days, with no involvement of the mucous membrane. Greater than 80% of his body surface area (BSA) was involved and a positive Nikolsky sign was present **(** **Figure 1A** **)**. Laboratory test results revealed an inflammatory syndrome with C-reactive protein values of 89.2 mg/L, erythrocyte sedimentation rates (ESR) of 50 mm/h and serum procalcitonin values of 0.200 ng/mL. Microbial cultures of cutaneous secretions and blood culture were both negative. Chest CT-scan showed emphysema and lung interstitial changes without inflammatory changes. There were no signs of internal organ involvement. Prior to the appearance of skin lesions, treatment involved only afatinib, and based on his detailed medical history and typical manifestation of the lesion, a diagnosis of TEN was made, with a SCORTEN score of three. Afatinib was immediately terminated, with treatment now consisting of an intravenous administration of 1.2 mg/kg/d methyl-prednisolone, which was then reduced to 1.0 mg/kg/d after three days. Seven days after this treatment, there was a darkening of the erythema and a partial regeneration of the skin was observed **(** **Figure 1B** **)**, but the patient suddenly lapsed into a coma. MRI imaging indicated edema around the intracranial metastatic focus and disappearance of the left ventricle. The patient’s family relinquished further treatment and the patient was voluntarily discharged.

## Discussion

In July 2013, afatinib was approved for use as a first-line treatment of metastatic NSCLC with EGFR mutations (4). This drug can irreversibly inhibit signaling from EGFR, HER2, ErbB3 and ErbB4 (5) and afatinib has proved beneficial in patients with acquired resistance to first-generation EGFR inhibitors. It has been reported that afatinib was associated with several grade three or worse adverse events, including diarrhea, rash or acne and fatigue, with no skin-related adverse drug reactions above grade three (6). In our case, the patient experienced TEN after five days of afatinib treatment, which represents a grade four cutaneous adverse effect.

In most cases, SJS/TEN is related to a drug reaction, and a rare and potentially life-threatening mucocutaneous reaction, characterized by blisters and generalized epidermolysis, can be accompanied with multiple system involvement. The most common causative drugs are anticonvulsants, antidepressants, sulfonamides, non-steroidal anti-inflammatory agents, anti-infectives and targeted drugs (7). The typical latency period from drug exposure to symptoms ranges from 4 to 28 days (8). The incidence of SJS/TEN is 1-6 per 1,000,000, with a mortality rate of approximately 15% (9), most commonly resulting from sepsis (10). The histopathology of lesions in SJS/TEN varies from keratinocyte apoptosis to epidermal necrosis, with a partial infiltration of perivascular lymphocytes, histiocytes, and a few eosinophils being observed in the dermis (7). No standardized treatment guidelines or recommendations currently exist for the treatment of SJS/TEN, with discontinuation of causative drugs, supportive care and prevention of infection all serving as protocols for the management of SJS/TEN. It is generally accepted that the culprit drug should be immediately stopped and alternative drug treatments initiated when severe cutaneous adverse reactions are present. For NSCLC patients who experienced SJS/TEN induced by afatinib, gefitinib could be an alternative treatment option (11). Drug desensitization should be considered when culprit drugs must be used under conditions where no alternative drugs are available and the effects of alternative drugs are unsatisfactory. While reports from many studies involving drug desensitization have shown these protocols to be safe and effective (12), such procedures are absolutely forbidden in cases with severe immunocytotoxic reactions, vasculitis and in severe cutaneous adverse reactions, like that observed in SJS/TEN.

Our patient manifested bulla formation and typical exfoliation of the epidermis without mucosal involvement while 80% of SJS/TEN cases generally involve two or more mucous membranes (8). Clinically, SJS/TEN needs to be differentiated from other infectious diseases, autoimmune bullous diseases and lupus erythematosus. For this reason, a chest CT scan and cultured cutaneous secretions were included to exclude mycoplasma pneumonia and a staphylococcal scalded skin syndrome. Skin biopsy and direct immunofluorescence were not performed as consent was not obtained. According to the algorithm of drug causality for epidermal necrolysis (ALDEN) (13), the probable culprit drug was identified as afatinib, with an ALDEN score of four. As TEN is life-threatening, afatinib was immediately stopped and supportive care, intravenous methyl-prednisolone (1.2 mg/kg/d), oral ebastine and desloratadine were initiated. Although controversy remains with regard to use of glucocorticoids in SJS/TEN patients, our patient benefitted from such therapy. His body temperature returned to normal in one day, progression of the skin rash ceased within three days and the dose of methyl-prednisolone was reduced to 1.0 mg/kg/d. The cutaneous lesions partially improved after seven days and no severe side effects were observed. There is some evidence indicating that a combination therapy consisting of corticosteroids and IVIg is associated with reduced mortality rates and significant abbreviations in recovery times of TEN patients (14). For economic reasons, our patient refused IVIg therapy. It has also been reported that administration of TNF-α inhibitors can serve as a rapid and effective treatment for SJS/TEN in some cases. As malignant tumors were present in our patient, TNF-α inhibitor treatment was not considered in this case. The patient developed hypoproteinemia on the seventh day of hospitalization, for which he received intravenous human albumin and then suddenly lapsed into a coma due to multiple brain metastases. The patient’s family relinquished further treatment and the patient was voluntarily discharged.

To our knowledge, there have only been four cases of SJS/TEN associated with afatinib in patients with NSCLC (**Table 1**) **(** 11, 15–17). All of these patients received corticosteroid treatment, with two receiving corticosteroid pulse therapy and one receiving corticosteroid pulse therapy combined with IVIg. Corticosteroids have anti-inflammatory and immunosuppressant properties that inhibit the production of inflammatory cytokines and antibodies, as well as that of keratinocyte apoptosis induced by T cells (18). Due to limited evidence and varied results obtained, controversies remain with regard to the use of corticosteroids in SJS/TEN. However, all patients receiving corticosteroids benefited from this therapy, with four patients showing a complete recovery, and the lesions observed in our patient showed a partial improvement, but unfortunately died within two weeks after discharge due to brain metastases. The latency period of responses in these four cases were all greater than thirty days, while that of our case was only five days. In general, latency periods of symptoms to drug exposure range from 4 to 28 days in SJS/TEN cases (8). It would appear that SJS/TEN induced by afatinib is more likely to show a longer latency period. In the epidermis, suprabasal layers and outer layers of hair follicles and EGFR expression occur predominantly in undifferentiated, proliferating keratinocytes (19). And, this EFGR has been shown to regulate normal keratinocyte proliferation, differentiation, migration and survival (20). A potential mechanism for afatinib-induced SJS/TEN might involve an irreversible inhibition of the EGFR signal pathway, thus affecting epidermis differentiation and re-epithelialization, ultimately leading to extensive epidermal involvement (21). However, typical responses to SJS/TEN consist of a delayed hypersensitivity reaction, where cytotoxic T cells produce granulysin and granzyme that result in the death of keratinocytes (8). Such differences might serve as an explanation for the variations in latency periods between typical versus afatinib-induced SJS/TEN. As the latency period observed in our case was consistent with that of typical SJS/TEN, we propose that a link may exist among their mechanisms. Further studies will be required to clarify the potential mechanisms of afatinib-induced SJS/TEN.

**Table 1:** Case report of SJS/TEN associated with afatinib.

References	Country	Disease	Sex	Age	latency period	Therapy	Outcome
(15)	Germany	SJS	Female	79	64 days	Steroids (1mg/kg/day) Topical steroids	Recovered
(16)	Japan	SJS	Female	73	2 months	Methylprednisolone (1000mg/day); Prednisolone (1mg/kg/day)	Recovered
(17)	Japan	SJS	Female	65	32 days	IVIg (0.4g/kg/day) Methylprednisolone (1000mg/day); Prednisolone (50 mg/day)	Recovered
(11)	Germany	SJS/TEN overlap	Female	60	32 days	Prednisolone (30mg/day) Cetirizine (10mg/day); Prednisolone (500mg/day)	Recovered
Our case	China	TEN	Male	62	5 days	Methylprednisolone (1.2mg/kg/day) Ebastine(20mg/day); Desloratadine(5mg/day)	Improved*

SJS, Stevens-Johnson syndrome; TEN, toxic epidermal necrolysis; IVIg, intravenous immune globulin; *, after one week of treatment, there was a partial improvement in the cutaneous lesions but the patient died of metastatic brain tumor.

However, limitations associated with this case report should be noted. For example, there is a lack of some laboratory assay findings, such as a skin biopsy, direct immunofluorescence and test results for autoantibody profiles, all of which would be useful in establishing a differential diagnosis. Moreover, although results from previous studies have suggested that specific human leukocyte antigen alleles may be associated with SJS/TEN (22), it was not possible for us to examine the specific human leukocyte antigen alleles in our patient as consent was not obtained.

## Conclusion

In conclusion, severe cutaneous adverse reactions, like SJS/TEN, may occur at any time during afatinib therapy. Accordingly, close monitoring of skin lesions in patients receiving afatinib is warranted.

## Data availability statement

The original contributions presented in the study are included in the article/supplementary material. Further inquiries can be directed to the corresponding author.

## Author contributions

WY wrote and edited of the original draft. WY and JN took part in the diagnosis and management of patient. ZW investigated previous case reports. YL reviewed and edited the manuscript. JN was involved in critical revision of the manuscript for important intellectual content. All authors contributed to the article and approved the submitted version.
